# Fas Activated Serine-Threonine Kinase Domains 2 (FASTKD2) mediates apoptosis of breast and prostate cancer cells through its novel FAST2 domain

**DOI:** 10.1186/1471-2407-14-852

**Published:** 2014-11-20

**Authors:** Sharmistha Das, Kay T Yeung, Muktar A Mahajan, Herbert H Samuels

**Affiliations:** Department of Biochemistry and Molecular Pharmacology, PHL 814, New York University School of Medicine, 455 First Ave., New York, NY 10016 USA

**Keywords:** Apoptosis, Breast and prostate cancer, NRIF3, DIF-1, IRF-2BP2, FASTKD2

## Abstract

**Background:**

Expression of NRIF3 (Nuclear Receptor Interacting Factor-3) rapidly and selectively leads to apoptosis of breast cancer cells. This occurs through binding of NRIF3 or its 30 amino acid Death Domain-1 (DD1) region to the transcriptional repressor, DIF-1 (DD1 Interacting Factor-1). DIF-1 acts in a wide variety of breast cancer cells but not other cell types to repress the pro-apoptotic gene, FASTKD2. Expression of NRIF3 or DD1 inactivates the DIF-1 repressor leading to rapid derepression of FASTKD2, which initiates apoptosis within 5–8 h of expression. Although FASTKD2 is an inner mitochondrial membrane protein, it does not require mitochondrial localization to initiate apoptosis.

**Methods:**

Androgen dependent LNCaP cells as well as two androgen independent LNCaP cell lines (LNCaP-AI and LNCaP-abl) were studied and LNCaP-AI cells were engineered to conditionally express DD1 or the inactive DD1-S28A with 4-hydroxytamoxifen. Apoptosis was assessed by TUNEL assay. FASTKD2 is related to 4 other proteins encoded in the human genome (FASTKD1, 3, 4, 5). All contain a poorly conserved putative bipartite kinase domain designated as FAST1_FAST2. We examined whether expression of any of the other FASTKD isoforms leads to apoptosis and sought to identify the region of FASTKD2 necessary to initiate the apoptotic pathway.

**Results:**

Of the FASTKD1-5 isoforms only expression of FASTKD2 leads to apoptosis. Although, the NRIF3/DD1/DIF-1 pathway does not mediate apoptosis of a wide variety of non-breast cancer cell lines, because of certain similarities and gene signatures between breast and prostate cancer we explored whether the NRIF3/DD1/DIF-1/FASTKD2 pathway mediates apoptosis of prostate cancer cells. We found that the pathway leads to apoptosis in LNCaP cells, including the two androgen-independent LNCaP cell lines that are generally resistant to apoptosis. Lastly, we identified that FASTKD2-mediated apoptosis is initiated by the 81 amino acid FAST2 region.

**Conclusions:**

The NRIF3/DIF-1/FASTKD2 pathway acts as a “death switch” in breast and prostate cancer cells. Deciphering how this pathway is regulated and how FASTKD2 initiates the apoptotic response will allow for the development of therapeutic agents for the treatment of androgen-independent prostate cancer or Tamoxifen-unresponsive Estrogen Receptor negative tumors as well as metastatic breast or prostate cancer.

## Background

Programmed cell death or apoptosis, a fundamental process in growth and development, can be targeted in the treatment of various tumors [1]. Several years ago we identified a nuclear hormone receptor co-activator which we refer to as Nuclear Receptor Interacting Factor 3 (NRIF3) [2]. Surprisingly we found that expression of NRIF3 rapidly leads to caspase-2-dependent apoptosis in a wide variety of Estrogen Receptor positive or negative human breast cancer cell lines (e.g. SKBR3, MCF-7, T-47D, MDA-435, MDA-231 and MDA-231/ER^+^) and two mouse breast cancer cell lines (4T1 and 67NR) [2–5]. However, NRIF3 expression did not lead to apoptosis in a wide variety of other cell types (e.g. U2OS, human osteosarcoma; 293, human kidney epithelium; UOK-145, kidney carcinoma; HepG2, human hepatoma, and HeLa, human cervical carcinoma) [2–4]. Apoptosis mediated by NRIF3 was documented by FACS analysis, binding of Annexin V, time-lapse imaging, and TUNEL assay [2, 3]. This apoptotic activity was mapped to a short ~30 amino acid region (amino acids 20–50) of NRIF3. We refer to this region as Death Domain-1 (DD1) since it is necessary and sufficient to mediate apoptosis of breast cancer cells. DD1 does not interact with nuclear receptors, thus, this apoptotic effect of NRIF3 is independent of its action as a nuclear receptor co-activator. Change of Ser28 to Ala28 (S28A) abrogates the ability of NRIF3/DD1 to mediate apoptosis suggesting that phosphorylation of Ser28 is important for this biological effect of NRIF3/DD1 [2, 3].

We cloned the intracellular target of NRIF3/DD1 and refer to this factor as DD1 Interacting Factor-1 (DIF-1) which is a transcriptional repressor [4]. Our studies indicated that DIF-1 (a.k.a IRF-2BP2) acts to selectively repress one or more pro-apoptotic genes in breast cancer cells (but not in the other cell types examined) and this repression is reversed by the binding of NRIF3/DD1 [4]. The notion that DIF-1 represses pro-apoptotic genes in breast cancer cells is further supported by the finding that knockdown of DIF-1 by siRNA leads to apoptosis of breast cancer cells but not of other cell types; including MCF-10A cells and C57MG cells, which are respectively immortalized normal human and mouse breast epithelial cell lines [4]. Thus, DIF-1 acts as a “death switch” whose activity can be attenuated by the binding of NRIF3/DD1 leading to pro-apoptotic gene expression in breast cancer cells [4].

Through microarray and expression studies, we identified FASTKD2 (Fas Activated Serine-Threonine Kinase Domains 2) as the pro-apoptotic target gene that is repressed by DIF-1 in breast cancer cells but not other cell types [5]. DIF-1 binds to the FASTKD2 gene in breast cancer cells but not to the FASTKD2 gene in other cell types (e.g. HeLa cells) [5]. Knockdown of FASTKD2 by siRNA prevents NRIF3/DD1-mediated apoptosis in breast cancer cells while expression of FASTKD2 leads to apoptosis in all cell types [5]. Our findings are consistent with a model where rapid and transient de-repression of the FASTKD2 gene in breast cancer cells leads to apoptosis [5].

Although, the NRIF3/DD1/DIF-1 pathway does not mediate apoptosis of a wide variety of non-breast cancer cell lines, because of certain similarities and gene signatures between breast and prostate cancer [6–8] we explored whether the NRIF3/DD1/FASTKD2 pathway mediates apoptosis of prostate cancer cell lines. We examined LNCaP cells which are androgen dependent (LNCaP-AD) [9] as well as two other LNCaP cell lines which express high levels of androgen receptor but are androgen independent with regard to growth (LNCaP-AI and LNCaP-abl) [10, 11]. Interestingly, LNCaP-AI and LNCaP-abl are much more resistant to apoptosis than androgen dependent LNCaP-AD cells [10, 11]. Here we report that all three LNCaP cell lines rapidly undergo apoptosis in response to NRIF3/DD1 through the rapid expression of the FASTKD2 gene. Moreover, we document that an 81 amino acid sequence in the putative FASTKD2 kinase domain region is sufficient to mediate apoptosis in LNCaP cells and other cell types.

## Methods

### Plasmids

pLPC-DD1-ERT2 or pLPC-DD1(S28A)-ERT2 retroviral based plasmids were described previously [5] and the expressed proteins are activated by by 4-hydroxytamoxifen (4-OHT). These vectors express a chimera with an N-Terminal FLAG epitope and a nuclear localization signal [5]. Full-length FASTKD2 generated by PCR and cloned into p3xFLAG-CMV-14 (Sigma) to yield FASTKD2 with a C-terminal 3xFLAG tag was described previously [5]. FASTKD2 lacking both the FAST kinase and the RAP domains [FASTKD2(1–455)] was generated by PCR and cloned into the EcoRI-KpnI site of p3xFLAG-CMV-14. DNA corresponding to the FAST2 domain (amino acids 538–619) and the FAST1_FAST2 region (amino acids 456–619) were generated by PCR and cloned into the EcoR1-KpnI site of pEGFP-C3. The number designations used are as described by Simarro et al. [12] although it has been suggested that Met 17 is the initiating codon [13]. All constructs were confirmed by sequencing. Vectors expressing pEGFP-DD1 and GAL4-DD1 and GAL4-DD1(S28A) and AIF-GFP were described previously [2]. YFP vectors expressing all five FASTKD proteins [12] were generously provided by Maria Simarro and Paul Anderson.

### Stable cell lines

LNCaP-AI cell lines stably expressing a DD1-ERT2 or a DD1(S28A)-ERT2 chimera were generated as previously described for T-47D, MCF-7 and SKBR3 breast cancer cells and HeLa cells [5]. In summary, 293T cells, seeded in 15-cm dishes at 5 million cells per dish, were transfected with ψA retroviral packaging vector and either pLPC-DD1-ERT2 or pLPC-DD1(S28A)-ERT2 by calcium phosphate precipitation. The retroviral supernatant was collected at 36 h and 60 h post-transfection. The supernatant was then filtered through a 0.45 um sterile filter and added to LNCaP-AI cells for infection. Forty-eight h post-infection, cells were selected through resistance to 2 ug/ml puromycin for two weeks. Single colonies of each of the stable cell lines were isolated by serial dilution and screened for the expression of DD1-ERT2 or DD1(S28A)-ERT2 by immunofluorescence using FLAG-M2 antibody (Sigma). Expression of DD1-ERT2, or DD1(S28A)-ERT2 in the isolated clones was also confirmed by FLAG-M2 Western blotting.

### Cell culture

All cell lines except HeLa were maintained in Dulbecco's modified Eagle's medium (DMEM) containing 10% fetal bovine serum (FBS) supplemented with glutamine and antibiotics. HeLa cells were maintained in DMEM containing 10% bovine calf serum supplemented with glutamine and antibiotics. Stable cell lines were maintained in DMEM-10% serum supplemented with glutamine and 2 ug/ml puromycin.

### siRNA transfection

siRNAs to knockdown FASTKD2 expression were obtained from Qiagen and were previously verified to knockdown FASTKD2 by over 90% [5]. The target sequence for FASTKD2 was ATGAATCACCGATCTCTTATA. A control siRNA contained four base changes. Cells were transfected with the siRNAs (40 nM) using HiPerfect siRNA transfection reagent (Qiagen) according to manufacturer's recommendation. To obtain efficient knockdown in LNCaP-AI cells, the cells were transfected with the siRNAs twice (on day 1 and day 2) and the cells were studied ~70 h after the initial tranfection.

### TUNEL assay

Cell lines were plated at a density of 30,000 cells per well on glass coverslips in 48-well tissue culture plates [4]. About 24 h later, the cells were transfected with the siRNA(s) as indicated using HiPerfect (Qiagen) or the indicated plasmids (50 ng) using Lipofectamine 2000 (Invitrogen). Generally, cells were usually harvested 15 h after plasmid transfection. The DD1-ERT2 and DD1(S28A)-ERT2 stable cell lines were examined between 5 to 15 h after the addition of 1 uM 4-OHT as indicated. Cells were washed three times with phosphate-buffered saline, fixed in 4% formaldehyde, and assayed for TUNEL using the *in situ* Cell Death Detection TMR red kit (Roche Diagnostics). Cells were then stained with 4',6-diamidino-2-phenylindole (DAPI) to visualize nuclei, mounted on slides, examined by fluorescent microscopy, and digitally photographed. Magnification bars are shown at the lower right of each TUNEL assay figure.

### Quantitative reverse transcription PCR (qRT-PCR)

qRT-PCR was carried out using total RNA extracted from cells using TRIzol (Invitrogen). One ug of RNA was treated with DNase1 (Fermentas), and reverse transcribed with random hexamers using a cDNA kit (Applied Biosystems) according to manufacturer's protocol. Specific PCR products were amplified using the FASTKD2 PCR primers [5] (forward primer, TCCTGAATCCCTAAACATGAAAA; reverse primer, GCCATAACTTCCACGAACTG), a 1:50 dilution of cDNA, and the Maxima SYBR Green/Fluorescein qPCR Master Mix (Fermentas). Forward and reverse primers for qRT-PCR of the other 4 FASTKD mRNAs (FASTKD1,3,4,5,) were as previously described [12]. SYBR green signals were measured in a BioRad iCycler machine. The values were normalized to an internal 18S ribosomal RNA control.

### Immunofluorescence

Cells were plated, treated, and fixed as described in the experiments for TUNEL assay. FLAG-M2 antibody (Sigma) and anti-mouse FITC antibody (Zymed) were used to stain for FLAG-DD1-ERT2 or FASTKD2-FLAG expression in fixed cells. After treatments and/or transfections, cells were fixed, and permeabilized with 1x PBS with 0.2% Triton-X100 for 10 min at 25°C. After 3 washes of 1x PBS, the cells were blocked with 3% BSA in 1x PBS for 45 min at 25°C, then incubated with 3 ug/ml of FLAG-M2 antibody (Sigma) in 3% BSA in 1x PBS. After the primary antibody incubation, the cells were washed three times in 1x PBS. The cells were then incubated with 7.5 ug/ml of the secondary anti-mouse FITC antibody (Zymed) for 1 h at 25°C. The cells were finally washed three times in 1x PBS, and stained with DAPI to visualize nuclei, mounted on slides, examined by fluorescent microscopy, and digitally imaged. Magnification bars are shown at the lower right of each figure.

## Results

### NRIF3/DD1 expression mediates apoptosis of LNCaP cells through activation of caspase-2 and an increase in mitochondrial permeability

In previous studies apoptosis mediated by NRIF3 in breast cancer cells was documented by FACS analysis, binding of Annexin V, time-lapse imaging, and TUNEL assay [2, 3]. In addition, evidance that NRIF3/DD1-mediated apoptosis in breast cancer cells involves caspase-2 comes from studes indicating that knockdown of caspase-2 expression transiently by siRNA [2] or stably with shRNA [3] abrogates the apoptotic response. Furthermore, zVAD-fmk did not block the apoptotic response while apoptosis was blocked with zVDVAD-fmk [2]. This is consistant with a role for caspase-2 in NRIF3/DD1-mediated apoptosis since zVAD-fmk is not a target of caspase-2 while zVDVAD-fmk exhibits a high affinity for caspase-2 [14]. Although zVDVAD-fmk can target caspase-3, evidence that caspase-3 is not essential for the apoptotic response comes from the finding that zVAD-fmk, which exhibits a high affinity for caspase-3, does not block NRIF3/DD1-mediated apoptosis [2]. Furthermore, NRIF3/DD1 mediates apoptosis in MCF-7 cells [2, 5] which do not express caspase-3 [15, 16].

**Figure 1:**
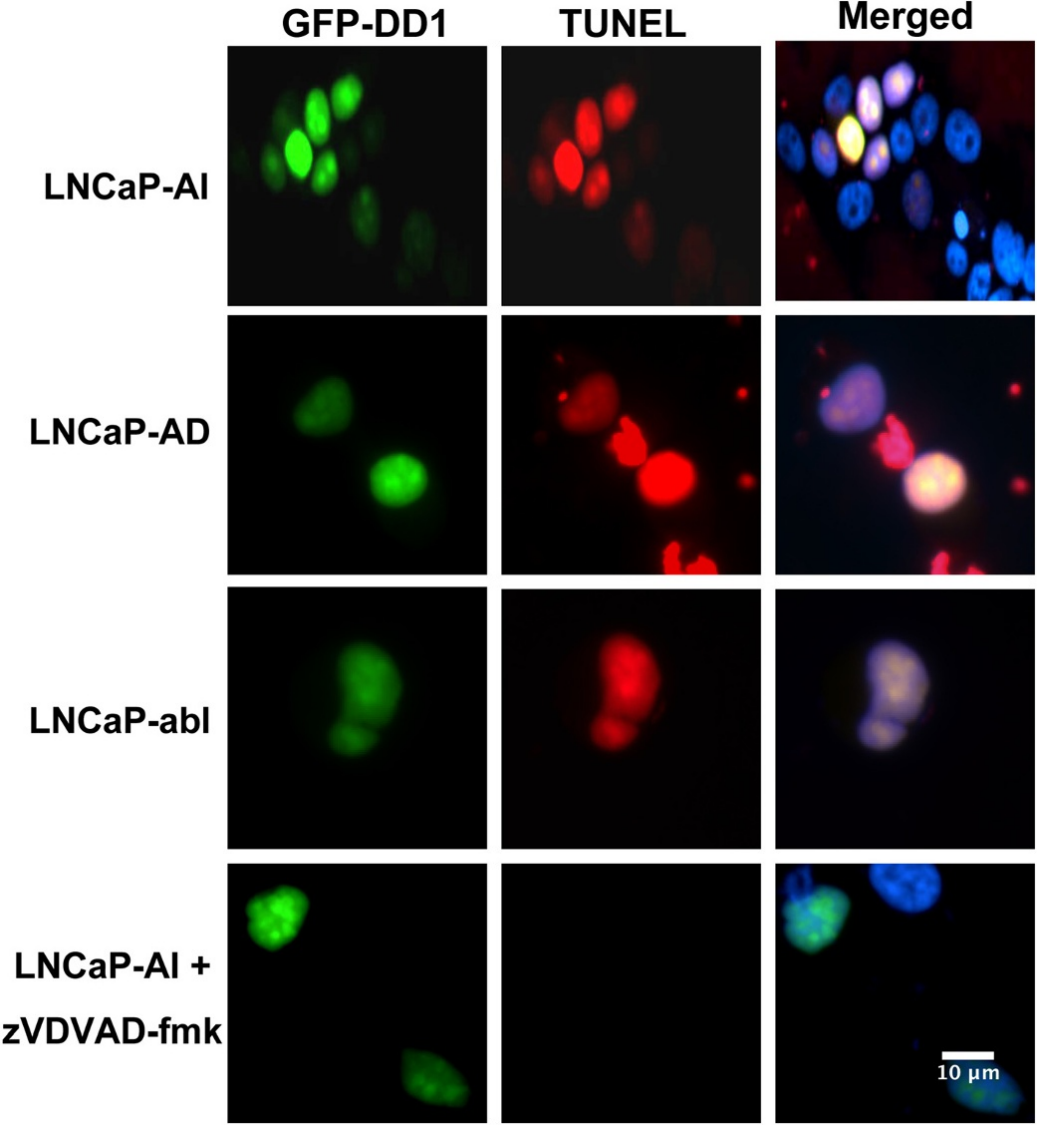
**Expression of DD1 leads to apoptosis in androgen dependent and androgen independent LNCaP cell lines.** LNCaP-AI, LNCaP-abl and LNCaP-AD cells were transfected with 50 ng of GFP-DD1 using Lipofectamine 2000. Fifteen h later the cells were fixed and permeablized for analysis of GFP fluorescence (green) or TUNEL assay (red). The Merged panel on the right also shows nuclei (blue) stained with DAPI. In the bottom panels LNCaP-AI cells were treated with 20 uM zVDVAD-fmk prior to transfection with of GFP-DD1.

To assess wether LNCaP cells undergo apoptosis by the same pathway as breast cancer cells, we expressed GFP-DD1 in LNCaP-AD, LNCaP-AI, and LNCaP-abl cells. Each cell line exhibited apoptosis in response to GFP-DD1 (TUNEL assay) (Figure 1). Expression of GFP-DD1(S28A) did not lead to apoptosis (not illustrated). In addition, zVDVAD-fmk blocks apoptosis mediated by DD1 in the prostate cancer cell lines (Figure 1). Like breast cancer cells zVAD-fmk was without effect (not shown). These findings in Figure 1 are identical to those found with a wide variety of breast cancer cell lines [4, 5] supporting the notion that the prostate cancer cell lines undergo NRIF3/DD1 mediated apoptosis through the same pathway. In addition, previous studies indicated that caspase-2-dependent apoptosis resulted from an increase in mitochondrial permeability [2, 17]. Such signaling of caspase-2 to mitochondria is thought to result from direct cleavage of the BH3-only protein BID which functions with Bax to increase mitochondrial permeability and release of factors that lead to apoptosis [17].

**Figure 2:**
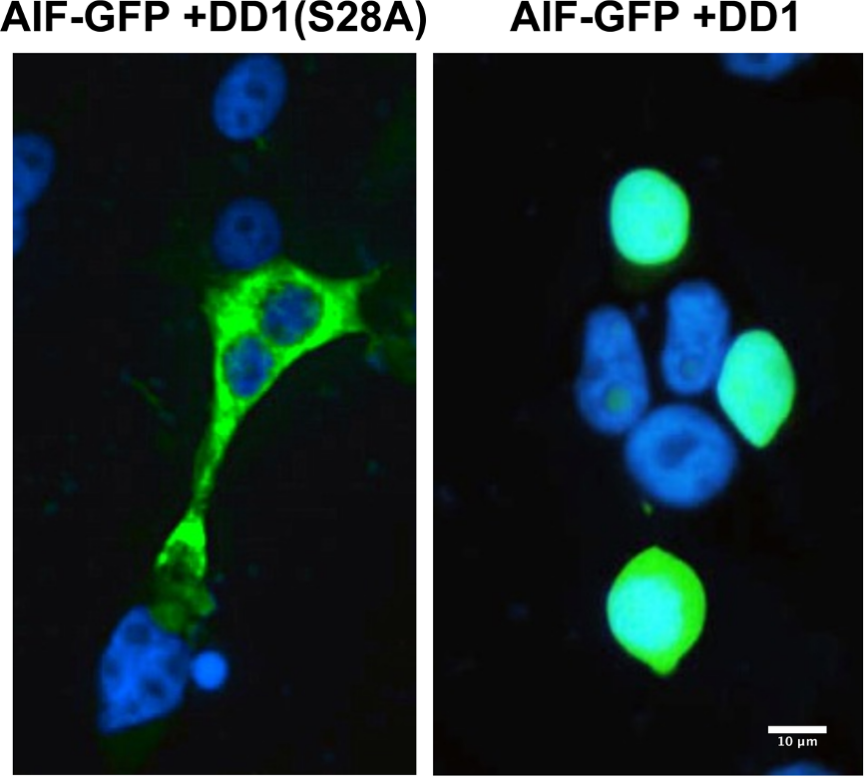
**DD1**-**mediated apoptosis in LNCaP cells leads to changes in mitochondrial membrane permeability and release of AIF which translocates to the cell nucleus.** LNCaP-AI cells were first transfected with a plasmid to express AIF-GFP. Twenty-four h later cells were transfected to express DD1 which leads to apoptosis, or DD1(S28A) which is inactive [DD1 and DD1(S28A) were expressed as GAL4 fusion proteins to ensure nuclear localization]. Fifteen h after the second transfection cells were fixed and permeablized for AIF-GFP fluorescence (green). Nuclei were stained with DAPI (blue). The Figure shows an extra-nuclear mitochondrial distribution of AIF-GFP in cells expressing DD1(S28A) while in cells expressing DD1, AIF-GFP is localized to the nucleus.

To further document that NRIF3/DD1-mediated apoptosis is associated with release of mitochondrial pro-apoptotic factors in LNCaP cells, we examined the cell distribution of AIF (Apoptosis Inducing Factor). AIF normally localizes to the inside of the outer mitochondrial membrane [18]. With changes in mitochondrial permeability AIF is released and localizes to the nucleus where it initiates DNA fragmentation [18]. To examine this we first transfected LNCaP-AI cells to express AIF-GFP. Twenty-four h after expression of AIF-GFP, cells were then transfected to express DD1 which leads to apoptosis, or DD1(S28A) which is inactive [DD1 and DD1(S28A) were expressed as GAL4 fusion proteins to ensure nuclear localization]. Figure 2 illustrates the cell distribution of AIF-GFP in cells where DD1(S28A) was expressed. AIF-GFP is localized completely outside of the nucleus (nuclei are stained with DAPI). In contrast, cells which express DD1 show complete nuclear localization of AIF-GFP consistent with a DD1-mediated effect leading to changes in mitochondrial permeability.

### Conditional expression of DD1 and DD1(S28A) in LNCaP cells

**Figure 3:**
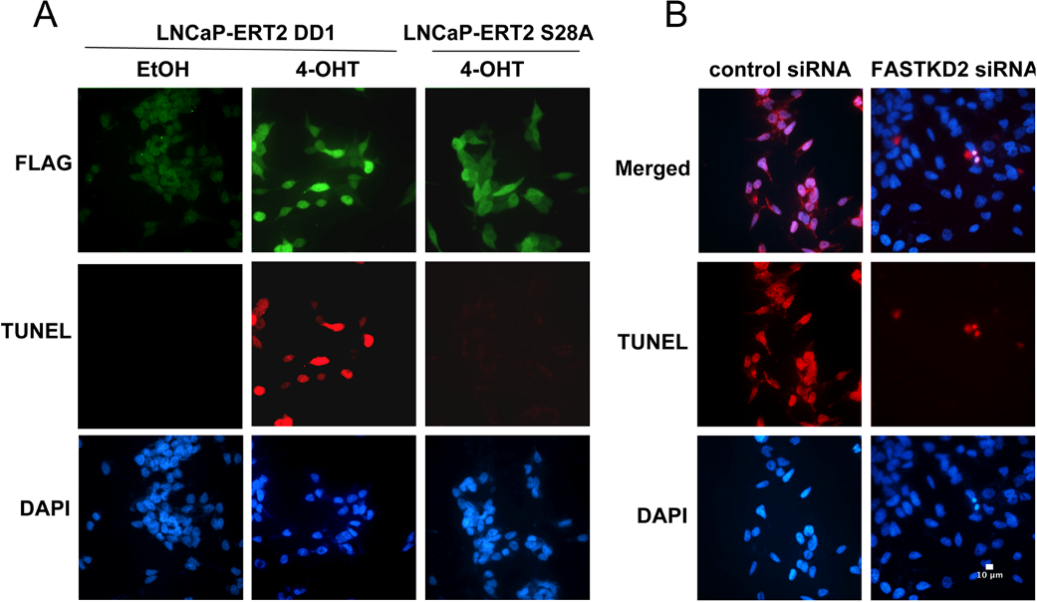
**Activation of DD1 leads to expression of FASTKD2 which mediates the apoptotic response. (A)** LNCaP-AI cells stably expressing DD1-ERT2 or DD1(S28A)-ERT2 were incubated with 1 uM 4-OHT for 8 h and then analyzed for DD1 and DD1(S28A) expression by immunofluorescense using FLAG-M2 antibody (green) and apoptosis by TUNEL assay (red). Nuclei were stained with DAPI (blue). **(B)** LNCaP-AI cells stably expressing DD1-ERT2 were transfected with a Control siRNA or an siRNA known to knock down expression of FASTKD2 [5]. Twenty-four h later the cells were transfected again with the siRNAs as described in the Methods section. Twenty h after the second siRNA transfection cells were incubated with 4-OHT for 15 h. Cells were then fixed and permeablized for TUNEL assay (red). Nuclei are stained with DAPI (blue). Cells treated with the FASTKD2 siRNA exhibited essentially no apoptosis after 4-OHT incubation while cells treated with the Control siRNA showed extensive apoptosis.

To further study the mechanism of DD1-mediated apoptosis of LNCaP cells, we generated stable LNCaP-AI cell lines expressing DD1-ERT2 or DD1(S28A)-ERT2. ERT2 is a mutated form of the human estrogen receptor-α ligand binding domain that does not bind estrogen agonists but binds the partial agonist–antagonist 4-hydroxytamoxifen (4-OHT) [19]. Both DD1-ERT2 and DD1(S28A)-ERT2 are expressed with an N-terminal nuclear localization signal and a FLAG epitope to allow for estimation of expression and cell distribution. Without 4-OHT, DD1-ERT2 and DD1(S28A)-ERT2 are sequestered in a heat shock protein complex mostly outside the nucleus. After 4-OHT incubation the chimeric proteins rapidly enter the nucleus. In addition, 4-OHT stabilizes the chimeric protein further increasing its level of expression in the cell. Such studies in breast cancer cells indicate that after 4-OHT incubation with DD1-ERT2 expressing cells, apoptosis (TUNEL) is detected within 4 h and is maximal between 5–10 h [5]. No apoptosis was found with the breast cancer lines expressing DD1(S28A)-ERT2 after 4-OHT incubation [5]. Figure 3A shows such a study in LNCaP-AI cells stably expressing DD1-ERT2 or DD1(S28A)-ERT2. Eight h after 4-OHT incubation the DD1-ERT2 LNCaP-AI cells exhibit extensive apoptosis by TUNEL assay while the DD1(S28A)-ERT2 LNCaP-AI cells are TUNEL negative.

### Role of FASTKD2 in Mediating Apoptosis by the NRIF3/DD1

Microarray studies with breast cancer cells expressing DD1-ERT2 or DD1(S28A)-ERT2 incubated with or without 4-OHT identified the FASTKD2 gene as the pro-apoptotic gene that is rapidly expressed when DIF-1 mediated repression is reversed by the binding of NRIF3/DD1 [5]. To establish that DD1-mediated apoptosis in the DD1-ERT2 LNCaP-AI cells is mediated by FASTKD2, we first transfected the cells with a control siRNA or an siRNA directed against FASTKD2 mRNA. To obtain efficient knockdown in LNCaP-AI cells, the cells were transfected with the siRNAs twice (on day 1 and day 2). Fifteen h after the second transfection cells were incubated with 4-OHT for 15 h and then examined for apoptosis by TUNEL assay (Figure 3B). Cells treated with the FASTKD2 siRNA were TUNEL negative while cells that received the control siRNA were TUNEL positive. Thus, like breast cancer cells, DD1-mediated apoptosis of LNCaP cells occurs through expression of the FASTKD2 gene.

**Figure 4:**
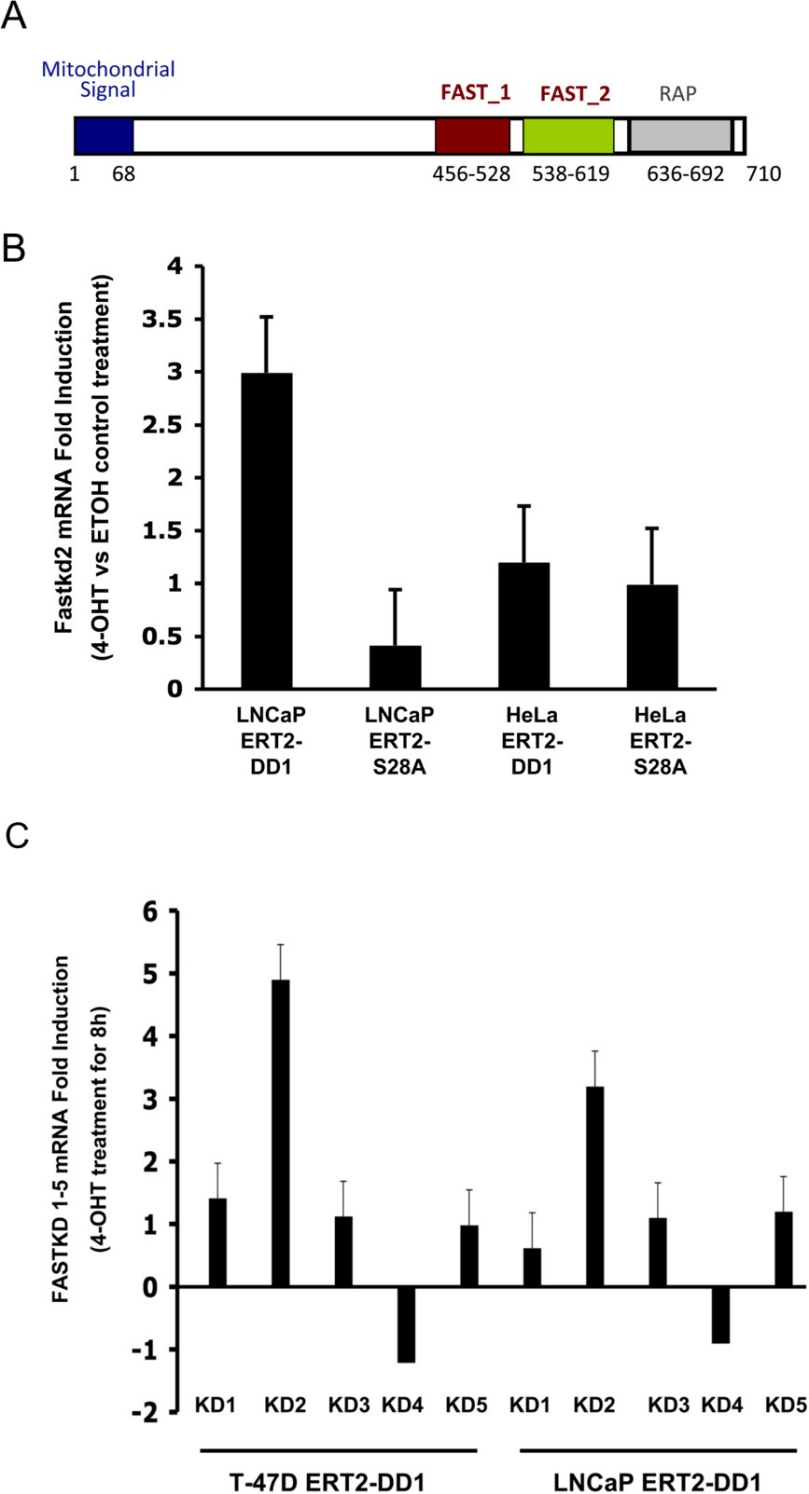
**FASTKD2 expression is enhanced by DD1 activation in LNCaP cells and is the only member of the FASTKD gene family that is enhanced by DD1. (A)** Domain structure of FASTKD2. **(B)** LNCaP-AI cells and HeLa cells stably expressing DD1-ERT2 or DD1(S28A)-ERT2 were first incubated with 20 uM zVDVAD-fmk to block apoptosis and then treated with 4-OHT (1 uM) or EtOH vehicle for 8 h. FASTKD2 mRNA expression was examined by qRT-PCR. Fold induction represents the FASTKD2 expression values of 4-OHT treated cells relative to those of EtOH treated cells. Data represents the mean +/- SEM from three representative experiments and the *p* values are given in the text. **(C)** LNCaP-AI and T-47D breast cancer cells stably expressing DD1-ERT2 were incubated with 4-OHT (1 uM) or EtOH vehicle for 8 h (cells were pretreated with 20 uM zVDVAD-fmk to block apoptosis). The expression of the 5 FASTKD mRNAs (indicated as KD1, KD2, KD3, KD4, and KD5) was examined by qRT-PCR using the primers indicated in the Methods section and in [12]. Fold induction represents the FASTKD expression values of 4-OHT treated cells relative to those of EtOH treated cells. Data represents the mean +/- SEM from three representative experiments and the *p* values are given in the text.

FASTKD2 is an inner mitochondrial membrane protein [13] and Figure 4A illustrates the domain organization of FASTKD2. The protein contains an N-terminal mitochondrial uptake signal, two putative FAST kinase-like domains (FAST1, FAST2) and a putative RNA-binding domain (RAP) near the C-terminus [12]. To study expression of the FASTKD2 gene, LNCaP-AI cells as well as HeLa cells stably expressing DD1-ERT2 and DD1(S28A)-ERT2 were incubated with 4-OHT or an EtOH vehicle control for 8 h followed by analysis of FASTKD2 mRNA abundance by quantitative qRT-PCR (Figure 4B). Prior to addition of 4-OHT, the cells received zVDVAD-fmk to block apoptosis to eliminate such an effect on the analysis. As previously found, FASTKD2 is not increased by DD1 in the two HeLa cell lines (*p* = 0.73) [5]. However, FASTKD2 mRNA levels were stimulated by 4-OHT in the DD1-ERT2 (*p* = 0.007) but not in the DD1(S28A)-ERT2 LNCaP-AI cells (*p* = 0.32).

Based on sequence homology FASTKD2 is related to 4 other human proteins (FASTKD 1,3,4,5) [12]. All five proteins localize to mitochondria and contain two putative FAST kinase-like domains (FAST1, FAST2) and a putative RNA-binding domain (RAP) near the C-terminus [12]. Although these proteins contain these FAST kinase-like domains which are related to the originally identified FASTK [20, 21], they have not been documented to exhibit kinase activity. In addition, the “kinase” domains do not contain conserved sequences typical of a kinase ATP binding site. Furthermore, alignment of the FAST1_FAST2 domains of FASTKD1-5 indicates that this region is about 20% similar/identical with a lot of gaps. Thus, it is not clear that these proteins mediate their effects through changes in phosphorylation. Although the biological functions of all these FAST kinase domain containing factors is not fully known, a recent study indicated that FASTKD3 influences basal and stress induced mitochondrial oxygen consumption [12]. To assess whether FASTKD genes other than FASTKD2 are regulated by NRIF3/DD1, we used qRT-PCR to study expression of all 5 FASTKD genes in both LNCaP-AI cells and T-47D breast cancer cells stably expressing DD1-ERT2. Figure 4C illustrates the results 8 h after 4-OHT incubation. Only FASTKD2 mRNA levels are increased in both DD1-ERT2 cell types (T-47D, *p* <0.001; LNCaP, *p* = 0.005) while we consistently note a slight reduction in FASTKD4 expression. A similar study with T-47D cells or LNCaP cells expressing DD1(S28A)-ERT2 showed no effect on any of the FASTKD mRNAs (not shown).

### Of the related FASTKD1-5 isoforms only FASTKD2 mediates apoptosis

**Figure 5:**
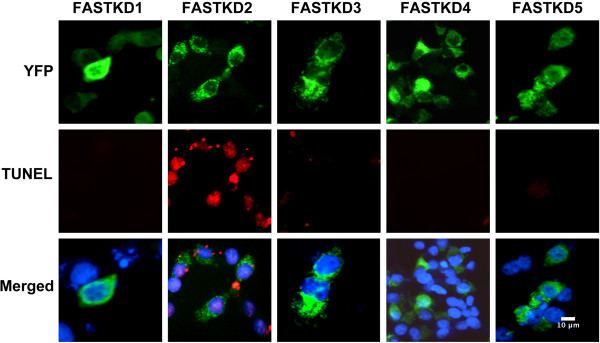
**Of the FASTKD isoforms only FASTKD2 mediates apoptosis.** Hela cells were transfected to express the 5 FASTKD isoforms as YFP chimeras with YFP at the C-terminus. Fifteen h later cells were fixed and permeablized for analysis of YFP fluorescence (green) and apoptosis by TUNEL assay (red). Nuclei were stained with DAPI (blue). The FASTKD-YFP chimeras localize in a peri-nuclear fashion which was previously documented to reflect mitochondrial localization [12]. Only expression of FASTKD2 leads to apoptosis.

To assess whether apoptosis mediated by FASTKD2 is unique or is found with all the FASTKD proteins we expressed all five FASTKD proteins as Yellow Fluorescent Protein (YFP) chimeras. Similar to the original study with these YFP chimeras [12] we found that all of the FASTKD proteins localize to the peri-nuclear region (Figure 5) which Simarro et al. have shown to be mitochondria [12]. However, when expressed in cells, only FASTKD2 leads to apoptosis in HeLa, T-47D, and LNCaP cells. Shown in Figure 5 is a representative study where apoptosis was assessed by TUNEL assay in HeLa cells. Fifteen h after expression of the FASTKD proteins, only cells expressing FASTKD2 are TUNEL positive.

### The FAST2 domain mediates the apoptotic effect of FASTKD2

**Figure 6:**
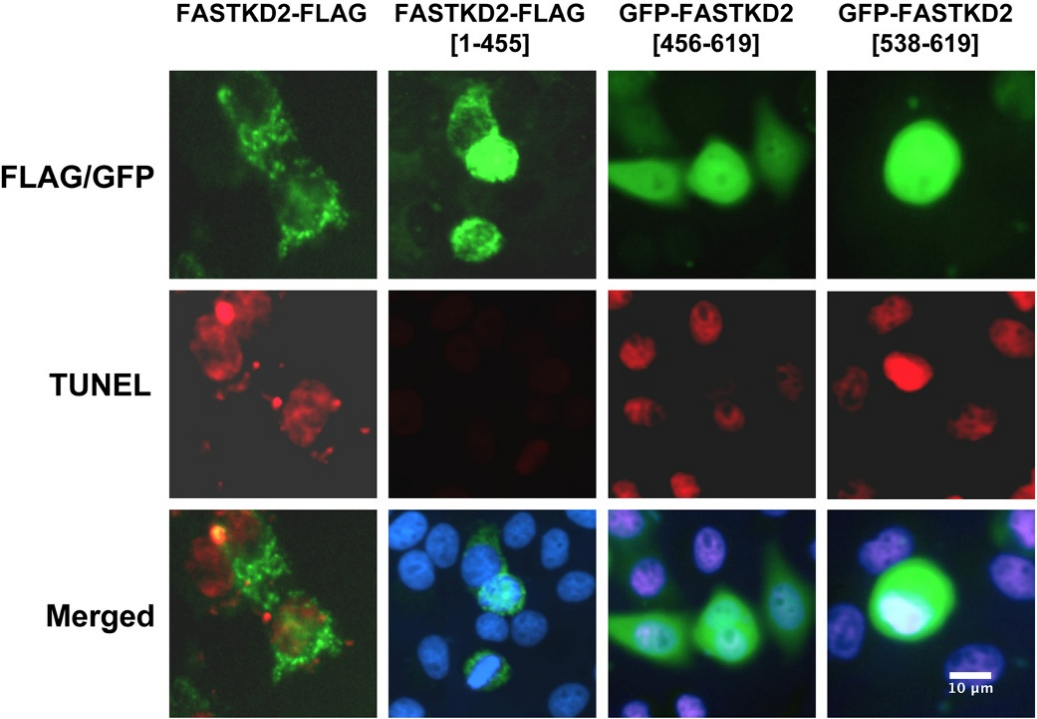
**The FAST2 domain mediates the apoptotic effect of FASTKD2.** HeLa cells were transfected with 50 ng of vectors that express either FASTKD2, FASTKD2(1–455) lacking the FASTKD2 FAST1_FAST2 and RAP domains, FASTKD2(456–619) which contains the FAST1_FAST2 domain or FASTKD2(538–619) which contains just the FAST2 domain. Fifteen h later cells were fixed and permeablized for TUNEL assay (red) and fluorescence studies (green). Nuclei were stained with DAPI (blue). FASTKD2 and FASTKD2(1–455) were detected by immunofluorescence using FLAG-M2 antibody while expression of the other FASTKD2 domains were detected as GFP chimeras.

Although FASTKD2 is an inner mitochondrial membrane protein, apoptosis mediated by FASTKD2 does not appear to require mitochondrial localization since expression of FASTKD2 lacking the N-terminal mitochondrial import signal (ΔM-FASTKD2) leads to apoptosis [5]. This suggests that when FASTKD2 is rapidly expressed, it acts to activate pro-apoptotic or inhibit anti-apoptotic factors on the surface or outside of mitochondria prior to import. To assess the domain of FASTKD2 that mediates apoptosis we generated vectors expressing the following regions of FASTKD2; amino acids 1–455 which contains residues N-terminal of the FAST kinase domains, amino acids 456–619 which contains the FAST1_FAST2 domains and amino acids 538–619 which contains just the FAST2 domain [12] (see Figure 4A). FASTKD2 and FASTKD2 (1–455) are expressed with a C-terminal FLAG tag while FASTKD2 (538–619) and FASTKD2 (456–619) were expressed with GFP at their N-termini. Figure 6 shows a representative study with these vectors in HeLa cells, although similar results were found for T-47D and LNCaP cells (not illustrated). Cells were transfected with the above vectors and 15 h later analyzed for FLAG/GFP expression and apoptosis by TUNEL assay. As previously reported, full-length FASTKD2 exhibits a peri-nuclear localization characteristic for mitochondria [12] and mediates apoptosis. FASTKD2 (1–455) containing the N-terminal mitochondrial import signal but lacking the FAST1_FAST2 domains exhibits a similar cell distribution as FASTKD2 but its expression did not lead to apoptosis. GFP-FASTKD2(456–619) and GFP-FASTKD2(538–619) are diffusely expressed in the cell and each resulted in apoptosis indicating that it is the 81 amino acid FAST2 domain of FASTKD2 that initiates the apoptotic cascade. Note that nearby cells not expressing GFP-FASTKD2(456–619) or GFP-FASTKD2(538–619) (Figure 6) also undergo apoptosis which is consistent with a bystander effect we previously reported for NRIF3/DD1-mediated apoptosis [5] which occurs through FASTKD2.

## Discussion

FASTKD2 with a nonsense mutation in both alleles on chromosome 2 was identified in a family with a transmitted Infantile Mitochondrial Encephalophy [13]. These individuals were shown to have a marked decrease in cytochrome c oxidase activity (Complex IV), which receives electrons from cytochrome c and transfers them to molecular oxygen [13]. FASTKD2 localizes to the inner mitochondrial compartment and is thought to be a component of Complex IV [13]. Fibroblasts from individuals with Infantile Mitochondrial Encephalophy show less apoptosis in response to Staurosporine [13]. In microarray studies we previously identified a rapid increase in expression of FASTKD2 in breast cancer cells expressing NRIF3/DD1 but no change in other cells types [5].

The FASTKD2 gene appears to be repressed by DIF-1 and the binding of NRIF3/DD1 leads to rapid de-repression of the FASTKD2 gene [5]. Interestingly, the other members of the FASTKD gene family are not enhanced through the NRIF3/DD1/DIF-1 pathway in breast cancer cells or LNCaP cells (Figure 4C) nor does their expression lead to apoptosis (Figure 5). In other cell types examined the FASTKD2 gene is not regulated by NRIF3/DD1 [5]. ChIP analysis indicated that DIF-1, and its related associated proteins IRF2BP1 and EAP-1, bind to the first untranslated exon of the FASTKD2 gene in breast cancer cells while DIF-1 does not bind to the gene in HeLa cells [5].

Since FASTKD2 is a highly pro-apoptotic factor, its expression and activity must be tightly controlled and regulated. Mitochondrial proteins encoded by nuclear genes are synthesized on free ribosomes and are thought to enter mitochondria directly through a pre-sequence; while other proteins with internal targeting signals associate with chaperones which target the mitochondrial import mechanism [22]. The mechanism of FASTKD2 mitochondrial import is not known but it does contain an N-terminal mitochondrial import signal which when removed prevents mitochondrial import [5, 13]. If FASTKD2 is directly imported *via* its pre-sequence, its expression needs be under tight control since over-expression can lead to apoptosis since mitochondrial import of certain proteins may not be an extremely rapid process [22]. In our studies, expression of NRIF3/DD1 leads to a rapid 3- to 7-fold increase in FASTKD2 expression within 5–8 h in breast cancer cell lines [5] as well as in LNCaP cells (Figure 4B and 4C). This rapid increase in FASTKD2 may not be rapidly imported into mitochondria and, thus, generate an extra-mitochondrial “threshold” level that is sufficient to spuriously initiate an apoptotic response. Consistent with that model is that expression of FASTKD2 without the mitochondrial import signal [5], or the FAST1_FAST2 region or just the FAST2 domain (Figure 6), which do not localize to mitochondria, leads to rapid apoptosis. Thus, the 81 amino acid FAST2 region mediates the pro-apoptotic effect of FASTKD2. As indicated in the results section, the FAST1_FAST2 domains do not contain conserved motifs typical of a kinase and the FAST1_FAST2 domains of the five FASTKD proteins are only about 20% similar/identical with significant gaps. Although we can’t exclude the possibility that FASTKD2 mediates its apoptotic response through phosphorylation, it is likely that it initiates apoptosis *via* a different mechanism.

Why NRIF3/DD1 regulates the FASTKD2 gene in breast cancer cells and LNCaP cells but not other cell types is currently unknown. For breast cancer cells we showed that the DIF-1 complex containing the related proteins IRF-2BP1 and EAP-1 binds to the 5’-untranslated exon of the FASTKD2 gene [5]. However, the DIF-1 complex does not bind to this region of the FASTKD2 gene in HeLa cells which expresses DIF-1, IRF2BP1 and EAP1 at similar levels as in breast cancer cells [5]. Mass spectrometry and silver stain gel studies indicate that the proteins associated with the DIF-1 complex differ in HeLa and T-47D breast cancer cells [5]. Thus, cells where FASTKD2 is regulated by DIF-1 may express components that allow for DIF-1 complex binding to the gene, possibly displacing regulatory factors that act to regulate low levels of expression of the gene in other cell types. Alternatively, cells where DIF-1 does not regulate the FASTKD2 gene, such as HeLa cells, may contain factors that interact with the DIF-1 complex preventing the complex from binding to and regulating the gene. In future mass spectrometry and functional studies we hope to explore such possible alternative models as well as identify the cellular target(s) of the FAST2 domain.

## Conclusions

The NRIF3/DD1/DIF-1/FASTKD2 pathway is a new pathway to therapeutically target Estrogen Receptor negative breast or androgen independent prostate cancer or metastatic cancer for therapy. In particular, in this study we found that expression of NRIF3/DD1 efficiently leads to apoptosis in LNCaP-AI and LNCaP-abl cells which are much more resistant to apoptosis than the parent LNCaP-AD cells [10, 11]. In previous studies we found that the 104 C-terminal amino acids of DIF-1 binds NRIF3/DD1 [4]. Future structural studies with this region of DIF-1 with a DD1-S28-phosphopeptide will hopefully lead to a crystal structure that provides a picture of the pharmacophore or chemical blueprint of the DD1/DIF-1 interaction. This can provide the structural basis for identifying small molecule DD1 mimetics that would be useful in targeting DIF-1 in breast and prostate cancer in animal models and in future clinical trials.

## Abbreviations

AIF: Apoptosis-inducing factor
DD1: Death Domain-1 region of NRIF3
DIF-1: DD1 interacting factor a.k.a. IRF-2BP2, interferon regulatory factor-2 binding protein-2
ERT2: Mutated estrogen receptor ligand binding domain activated by 4-OHT
FASTKD2: Fas Activated Serine-Threonine Kinase Domains 2
NRIF3: Nuclear receptor interacting factor-3
4-OHT: 4-hydroxytamoxifen
zVDVZD-fmk: Benzyloxycarbonyl-Val-Asp(OMe)-Val-Ala-Asp(OMe)-fluoromethylketone.
